# Patterns of Fetal and Infant Growth and Brain Morphology at Age 10 Years

**DOI:** 10.1001/jamanetworkopen.2021.38214

**Published:** 2021-12-09

**Authors:** Carolina C. V. Silva, Hanan El Marroun, Sara Sammallahti, Meike W. Vernooij, Ryan L. Muetzel, Susana Santos, Vincent W. V. Jaddoe

**Affiliations:** 1 The Generation R Study Group, Erasmus MC, University Medical Center Rotterdam, Rotterdam, the Netherlands; 2 Department of Pediatrics, Erasmus MC–Sophia Children’s Hospital, University Medical Center Rotterdam, Rotterdam, the Netherlands; 3 Department of Child and Adolescent Psychiatry/Psychology, Erasmus MC, University Medical Center Rotterdam, Rotterdam, the Netherlands; 4 Department of Psychology, Education and Child Studies, Erasmus University Rotterdam, Rotterdam, the Netherlands; 5 Department of Radiology and Nuclear Medicine, Erasmus MC, University Medical Center Rotterdam, Rotterdam, the Netherlands; 6 Department of Epidemiology, Erasmus MC, University Medical Center Rotterdam, Rotterdam, the Netherlands

## Abstract

**Importance:**

Preterm birth and low birth weight are associated with brain developmental and neurocognitive outcomes in childhood; however, not much is known about the specific critical periods in fetal life and infancy for these outcomes.

**Objective:**

To examine the associations of fetal and infant growth patterns with brain morphology in children at school age.

**Design, Setting, and Participants:**

This population-based, prospective cohort study was conducted from February 1 to April 16, 2021, as a part of the Generation R Study in Rotterdam, the Netherlands. The study included 3098 singleton children born between April 1, 2002, and January 31, 2006.

**Exposures:**

Fetal weight was estimated in the second and third trimesters of pregnancy by ultrasonography. Infant weight was measured at birth and at 6, 12, and 24 months. Fetal and infant weight acceleration or deceleration were defined as a change in SD scores greater than 0.67 between time points. Infant measurements also included peak weight velocity, and age and body mass index reached at adiposity peak.

**Main Outcomes and Measures:**

Brain structure, including global and regional brain volumes, was quantified by magnetic resonance imaging at age 10 years.

**Results:**

The study evaluated 3098 children (mean [SD] age at follow-up, 10.1 [0.6] years; 1557 girls [50.3%]; and 1753 Dutch [57.8%]). One SD score–higher weight gain until the second and third trimesters, birth, and 6, 12, and 24 months was associated with larger total brain volume independently of growth during any other age windows (second trimester: 5.7 cm^3^; 95% CI, 1.2-10.2 cm^3^; third trimester: 15.3 cm^3^; 95% CI, 11.0-19.6 cm^3^; birth: 20.8 cm^3^; 95% CI, 16.4-25.1 cm^3^; 6 months: 15.6 cm^3^; 95% CI, 11.2-19.9 cm^3^; 12 months: 11.3 cm^3^; 95% CI, 7.0-15.6 cm^3^; and 24 months: 11.1 cm^3^; 95% CI, 6.8-15.4 cm^3^). Compared with children with normal fetal and infant growth, those with fetal and infant growth deceleration had the smallest total brain volume (–32.5 cm^3^; 95% CI, –53.2 to –11.9 cm^3^). Children with fetal weight deceleration followed by infant catch-up growth had similar brain volumes as children with normal growth. Higher peak weight velocity and body mass index reached at adiposity peak were associated with larger brain volumes. Similar results were observed for cerebral and cerebellar gray and white matter volumes.

**Conclusions and Relevance:**

This cohort study’s findings suggest that both fetal and infant weight growth might be critical for cerebral and cerebellar brain volumes during childhood. Whether these associations link to neurocognitive outcomes should be further studied.

## Key Points

**Question:**

Are fetal and infant weight growth associated with cerebral and cerebellar gray and white matter volumes at age 10 years?

**Findings:**

In this population-based cohort study of 3098 children, increased weight gain during the fetal period and the first 2 years of life was associated with larger brain volumes at age 10 years. Compared with children with normal fetal and infant growth, those who experienced fetal weight deceleration followed by infant catch-up growth had similar brain volumes later in childhood.

**Meaning:**

The findings of this study suggest that early-life growth patterns may be associated with cerebral and cerebellar brain volumes in children at school age.

## Teaser

This cohort study examines the association of fetal and infant grown patters with brain morphology at school age among children in the Netherlands.

## Introduction

Fetal life and infancy are critical periods for human brain development.^1,2^ During fetal life, and especially in the third trimester of gestation, there is an important growth in brain size. The growth rates of the cortical gray matter and cerebellum peak during this period.^3,4^ Cortical gray matter volume also increases up to 150% in the first year of postnatal life and up to 20% in the second year.^5,6^ Adverse experiences occurring during these periods may permanently influence brain structure and function.^7,8,9^ Currently, evidence to support this hypothesis is mainly based on studies showing that children born preterm or with low birth weight are at risk for suboptimal neurodevelopmental outcomes.^10,11,12,13,14,15,16,17,18,19^ However, gestational age and weight at birth are merely the end point of fetal development and the starting point for infancy. In infancy, children born small for gestational age (SGA), appropriate for gestational age (AGA), and large for gestational age (LGA) have different growth patterns.^20^ Children born SGA tend to have catch-up growth, whereas those born LGA tend to have infant growth deceleration.^20,21^ Observational studies suggest that rapid weight gain in infancy is associated with benefits to later neurocognitive functioning, especially among those born preterm or SGA; however, little is known about its association with brain morphology.^22,23,24^ Population-based studies on the associations of fetal and infant growth patterns with brain structure enable identification of windows of vulnerability for brain development.

We hypothesized that early-life growth patterns might be associated with childhood brain morphology. In a large, population-based, prospective cohort study among 3098 children, we examined the associations of fetal and infant growth with global and regional brain volumes, measured by magnetic resonance imaging (MRI) at age 10 years.

## Methods

### Study Design

This cohort study was embedded in the Generation R Study, a population-based prospective cohort study in Rotterdam, the Netherlands. Pregnant women residing in the study area with a delivery date between April 1, 2002 ,and January 31, 2006, were invited to participate. Information on follow-up has been previously described.^25^ Written informed consent was obtained for all participants. The medical ethics committee of Erasmus Medical Center approved the study. This study followed the Strengthening the Reporting of Observational Studies in Epidemiology (STROBE) reporting guideline. We had information on fetal or infant growth in 9494 singleton births. Of these, 5704 children visited the research center at age 10 years. All children visiting the center were invited to undergo brain MRI. In total, 3882 children underwent neuroimaging assessment. Analyses were conducted for 3098 children for whom we had good-quality neuroimaging (eFigure 1 in the Supplement).

### Fetal and Infant Growth Measures

Fetal ultrasonography examinations were performed in each trimester of pregnancy.^25,26^ Gestational age was established by first-trimester ultrasonography.^27^ Head circumference, abdominal circumference, and femur length were measured by second- and third-trimester ultrasonography. Fetal weight was estimated using the formula by Hadlock et al.^28^ Gestational age–adjusted SD scores were calculated using reference growth curves derived from the same cohort as the current study.^26^ At birth, gestational age was categorized into preterm (<37 weeks), term (37-42 weeks), or postterm (>42 weeks).^13^ Birth weight was obtained from medical records and was categorized into low (<2500 g), normal (2500-4500 g), or high (>4500 g).^29^ We created sex- and gestational age–adjusted SD scores for weight at birth within our study population by using the Growth Analyzer 3.5 (Dutch Growth Research Foundation), based on North European reference charts.^30^ Children born SGA and LGA were defined as gestational age– and sex-adjusted SD scores for birth weight below the 10th percentile and above the 90th percentile, respectively.^31^

Infant weight was measured in community health centers using a mechanical personal scale at approximately 6, 12, and 24 months. Age- and sex-adjusted SD scores were obtained using Dutch reference growth charts.^32^

We categorized fetal and infant weight change into 3 groups (growth deceleration, normal growth, and growth acceleration) and created combined variables that reflect 9 different growth patterns. Fetal weight change was defined as growth between the second trimester and birth. Infant weight change was defined as growth from birth to 24 months in 69.4% of the full group. To achieve adequate sample size on subgroup analyses, if weight at 24 months was missing, we calculated the weight change by using weight at 12 or 6 months (20% and 10.6% of the full group, respectively).^31^ Growth acceleration or deceleration were defined as a change in SD scores greater than 0.67 between time points. This clinically significant change reflects the difference between 2 percentile lines on the growth charts.^33^

Peak weight velocity, reflecting the greatest weight growth in infancy, was derived using the Reed1 model by sex on all weight measurements taken from birth to 3 years of age, including birth weight.^34,35^ To obtain age and body mass index (BMI; calculated as weight in kilograms divided by height in meters squared) reached at adiposity peak, we analyzed repeated infant BMI measurements using a cubic mixed-effects model fitted on a log(BMI) scale from age 14 days to 1.5 years, adjusted for sex, which showed the best fit to the data.^35,36^ The growth measures are presented in detail in eTable 1 in the Supplement.

### Brain Outcomes

During the study visit at 10 years, head circumference was measured according to standardized procedures.^25^ Brain imaging acquisition has been described previously.^37^ Briefly, all images were acquired using the same scan protocol on a single 3-Tesla (T) scanner (Discovery MR750, GE Healthcare) using an 8-channel head coil. A high-resolution 3-dimensional T1-weighted structural scan was obtained with an inversion recovery fast-spoiled gradient recalled sequence. Parameters were as follows: repetition time = 8.77 ms; echo time = 3.4 ms; inversion time = 600 ms; flip angle = 10°; field of view = 220 × 220 mm; acquisition matrix = 220 × 220; slice thickness = 1 mm; and number of slices = 230. At the scanner, T1 image quality was rated using a 6-point Likert scale: unusable, poor, fair, good, very good, and excellent. If the initial T1-weighted scan was rated as unusable or poor, the T1 sequence was repeated.^37^ Images were processed using FreeSurfer, version 6.0 (Florida State University Research Computing Center). Global and regional volumes, including total brain and cerebral and cerebellar gray and white matter volumes, were extracted. FreeSurfer procedures had good test-retest reliability across scanners.^38^ The quality of FreeSurfer output was visually inspected, and all scans rated as unusable (mostly due to motion) were excluded.^39^ Scans were reviewed by radiologists for the presence of incidental findings.^40^

### Covariates

Information on maternal characteristics, including age, ethnicity, educational level, family income, prepregnancy BMI, smoking, alcohol consumption, and folic acid supplement use, were obtained from questionnaires during pregnancy. Child sex and age at neuroimaging assessment, available from medical records, were included to account for the association of these variables with brain measurements. Intracranial volume was obtained from MRI scans. We measured BMI at 10 years, using height and weight measured without shoes and heavy clothing. Age- and sex-adjusted SD scores for BMI were obtained with Dutch reference growth charts (Growth Analyzer 4.0).^41^

### Statistical Analysis

First, participants were compared to nonparticipants to assess possible bias due to loss to follow-up. Second, we assessed the associations between the birth outcomes of gestational age and weight and size for gestational age and brain volumes at 10 years using multiple linear regression models. Third, we performed conditional linear regression analysis to identify independent critical periods for early-life growth associated with brain outcomes. Conditional regression analyses take into account correlations between early life growth measures at different ages.^42,43^ We regressed weight at each time point on weight at all previous time points and saved the residual scores, creating noncorrelated growth variables that reflect growth during a certain time period independently of growth during all previous time periods.^44^ Fourth, we used similar regression models to assess the associations of fetal and infant growth patterns, peak weight velocity, and BMI and age at adiposity peak with brain volumes at 10 years.

We used a hierarchical approach to examine the associations of fetal and infant growth with brain outcomes. If an association with a subcortical volume was observed, we performed post hoc analyses of the specific subcortical structures adjusted for intracranial volume. Nonlinearity of the association was assessed using generalized additive models and ruled out. For all analyses, we present a basic model including child sex and age at outcome, a confounder model, and a BMI model. Potential confounders were represented in a directed acyclic graph (eFigure 2 in the Supplement). Confounder models additionally included maternal age at intake, ethnicity, prepregnancy BMI, educational level, family income, smoking, alcohol consumption, and folic acid use during pregnancy. The BMI model additionally included childhood sex- and age-adjusted BMI SD scores at 10 years. We tested for statistical interactions by child sex in the above analyses, but none of these were significant.^45^ Because results were similar when we excluded children born preterm and with low birth weight, we did not restrict or stratify on birth outcomes. Missing data in covariates were multiple-imputed using a Markov chain Monte Carlo approach.^46^ In total, 30 imputed data sets were created and analyzed, presenting pooled effect estimates with 95% CIs. No correction for multiple testing was performed, as all exposures and outcomes were highly correlated among each other, and thus a small number of independent tests was expected.^47^ Participants were compared with nonparticipnts by using Pearson χ^2^ tests, independent-sample *t* tests, and Mann-Whitney tests. Statistical significance was defined as *P* < .05 (2-sided). Statistical analyses were performed using the Statistical Package of Social Sciences, version 25.0 for Windows (IBM Inc).

## Results

### Participant Characteristics

The study evaluated 3098 children (mean [SD] age, 10.1 [0.6] years; 1557 girls [50.3%] and 1541 boys [49.7%]; and 1753 Dutch [57.8%], 1026 non-Dutch and non-Western [33.8%], and 253 non-Dutch and Western [8.3%]) (Table 1). Nonresponse analyses showed that compared with mothers of children without brain MRI data available, mothers of children with brain MRI measurements were slightly older, were more likely to be of Dutch origin, were highly educated, and smoked less often during pregnancy (eTable 2 in the Supplement).

**Table 1.** Participant Characteristics^a^

Characteristic	No. (%) (N = 3098)
Maternal characteristics	
Age at intake, mean (SD), y	31.1 (4.9)
Ethnicity, No. (%) (N = 3032)	
Dutch	1753 (57.8)
Non-Dutch, Western	253 (8.3)
Non-Dutch, Non-Western	1026 (33.8)
Prepregnancy body mass index, median (95% range)^b^	22.5 (18.0-34.8)
Education, No. (%) (N = 2848)	
Primary school	186 (6.5)
Secondary school	1156 (40.6)
Higher education	1506 (52.9)
Monthly household income, US dollars (N = 2409)	
<1200	330 (13.7)
1200-2000	380 (15.8)
>2000	1699 (70.5)
Folic acid used, No. (%) (N = 2173)	1737 (79.9)
Alcohol consumption, No. (%) (N = 2449)	1238 (50.6)
Smoking, No. (%) (N = 2450)	501 (20.4)
Fetal characteristics	
Second trimester, median (95% range)	
Gestational age, wk	20.5 (18.7-23.3)
Estimated fetal weight, g	362 (248-612)
Third trimester, median (95% range)	
Gestational age, wk	30.4 (28.5-32.7)
Estimated fetal weight, g	1602 (1186-2150)
Birth characteristics	
Gestational age at birth, median (95% range), wk	40.1 (36.0-42.3)
Sex, No. (%)	
Female	1557 (50.3)
Male	1541 (49.7)
Birth weight, mean (SD), g	3446 (553)
Infant characteristics	
At 6 mo, median (95% range)	
Age at visit, mo	6.2 (5.2-7.9)
Weight, kg	7.8 (6.2-9.7)
At 12 mo, median (95% range)	
Age at visit, mo	11.0 (10.1-13.0)
Weight, kg	9.6 (7.7-11.8)
At 24 mo, median (95% range)	
Age at visit, mo	24.8 (23.4-28.1)
Weight, kg	12.8 (10.2-16.1)
Peak weight velocity, mean (SD), kg/y	12.1 (2.1)
Body mass index at adiposity peak, mean (SD)^b^	17.6 (0.8)
Age at adiposity peak, median (95% range), mo	8.4 (7.8-9.6)
Child characteristics	
Age at MRI, mean (SD), y	10.1 (0.6)
Head circumference, mean (SD), cm	53.0 (1.6)
Length, mean (SD), cm	141.6 (6.6)
Weight, median (95% range), kg	33.8 (24.4-53.0)
Body mass index, median (95% range)^b^	16.9 (14.1-24.3)

Abbreviation: MRI, magnetic resonance imaging.

^a^ Values are means (SDs), medians (95% range), or numbers of individuals. Participant characteristics are based on observed, not imputed, data (valid %).

^b^ Calculated as weight in kilograms divided by height in meters squared.

### Birth Outcomes

Table 2 shows positive associations of gestational age, weight, and size for gestational age at birth with childhood head circumference and brain volumes at 10 years. Compared with children born at term, those born preterm had smaller total brain volume (–28.4 cm^3^; 95% CI, –43.9 to –12.8 cm^3^). Compared with children with a normal weight at birth, those with low birth weight had smaller total brain volume (–44.9 cm^3^; 95% CI, –61.1 to –28.7 cm^3^). Compared with children born AGA, those born SGA had smaller total brain volume (–36.6 cm^3^; 95% CI, –47.4 to –25.9 cm^3^). Similar results were observed for subcortical gray, cerebral gray and white, and cerebellar gray and white matter volumes. After additional adjustment for childhood BMI, results remained significant (eTable 3 in the Supplement). Post hoc analyses showed that higher birth weight and size for gestational age were both associated with larger globus pallidus and nucleus accumbens volumes (eTable 4 in the Supplement).

**Table 2.** Associations of Birth Outcomes With Childhood Brain Outcomes

Birth outcomes	No.	Difference (95% CI)^a^						
		Head circumference (cm)	Total brain volume (cm^3^)	Cerebral gray matter volume (cm^3^)	Cerebral white matter volume (cm^3^)	Cerebellar gray matter volume (cm^3^)	Cerebellar white matter volume (cm^3^)	Subcortical gray matter volume (cm^3^)
Gestational age, wk		0.1 (0.0 to 0.1)^b^	4.7 (2.9 to 6.5)^b^	2.6 (1.7 to 3.5)^b^	1.2 (0.4 to 2.1)^b^	0.4 (0.2 to 0.6)^b^	0.2 (0.1 to 0.2)^b^	0.3 (0.2 to 0.4)^b^
<37	138	–0.3 (–0.5 to –0.0)^c^	–28.4 (–43.9 to –12.8)^b^	–16.6 (–24.3 to –8.9)^b^	–6.5 (–13.7 to 0.7)	–2.8 (–4.4 to –1.1)^b^	–0.8 (–1.3 to –0.4)^b^	–1.5 (–2.2 to –0.8)^b^
37-42	2718	[Reference]	[Reference]	[Reference]	[Reference]	[Reference]	[Reference]	[Reference]
>42	223	0.3 (0.1 to 0.5)^b^	10.8 (–1.6 to 23.3)	7.1 (0.9 to 13.3)^c^	1.6 (–4.2 to 7.3)	0.8 (–0.5 to 2.1)	0.3 (–0.0 to 0.7)	0.9 (0.4 to 1.5)^b^
Birth weight, 500 g		0.3 (0.3 to 0.4)^b^	20.3 (17.3 to 23.2)^b^	9.8 (8.4 to 11.3)^b^	7.8 (6.4 to 9.1)^b^	1.4 (1.0 to 1.7)^b^	0.5 (0.4 to 0.6)^b^	0.8 (0.7 to 1.0)^b^
<2500	126	–0.7 (–1.0 to –0.4)^b^	–44.9 (–61.1 to –28.7)^b^	–21.0 (–29.0 to –13.0)^b^	–16.4 (–23.9 to –8.9)^b^	–3.6 (–5.3 to –1.8)^b^	–1.6 (–2.1 to –1.1)^b^	–2.2 (–2.9 to –1.5)^b^
2500-4500	2890	[Reference]	[Reference]	[Reference]	[Reference]	[Reference]	[Reference]	[Reference]
>4500	78	0.7 (0.3 to 1.0)^b^	39.5 (19.1 to 60.0)^b^	18.7 (8.6 to 28.8)^b^	17.2 (7.8 to 26.6)^b^	1.5 (–0.7 to 3.7)	0.5 (–0.1 to 1.2)	1.6 (0.6 to 2.5)^b^
Size for gestational age, SD score		0.4 (0.3 to 0.4)^b^	21.9 (18.7 to 25.1)^b^	10.2 (8.6 to 11.8)^b^	9.0 (7.6 to 10.5)^b^	1.4 (1.0 to 1.7)^b^	0.4 (0.3 to 0.5)^b^	0.8 (0.7 to 10.0)^b^
Small (<10 percentile)	307	–0.6 (–0.8 to –0.5)^b^	–36.6 (–47.4 to –25.9)^b^	–16.7 (–22.0 to –11.3)^b^	–15.5 (–20.5 to –10.5)^b^	–2.2 (–3.4 to –1.1)^b^	–0.8 (–1.1 to –0.5)^b^	–1.4 (–1.8 to –0.9)^b^
Appropriate (10-90 percentile)	2458	[Reference]	[Reference]	[Reference]	[Reference]	[Reference]	[Reference]	[Reference]
Large (>90 percentile)	307	0.6 (0.4 to 0.8)^b^	34.4 (23.6 to 45.1)^b^	14.8 (9.4 to 20.1)^b^	15.3 (10.3 to 20.3)^b^	2.2 (1.1 to 3.4)^b^	0.7 (0.4 to 1.0)^b^	1.3 (0.9 to 1.8)^b^

^a^ Values are linear regression coefficients (95% CIs) and reflect the change in centimeters of childhood head circumference and in cubic centimeters of childhood brain structures for birth outcomes. Models are adjusted for child sex and age at the neuroimaging assessment, family income, and maternal age at intake, ethnicity, prepregnancy body mass index, educational level, smoking, alcohol use, and folic acid use during pregnancy.

^b^ *P* < .01.

^c^ *P* < .05.

### Critical Periods

Table 3 shows that increased weight gain during all of the examined time periods was associated with larger head circumference and total brain volume at 10 years of age. One SD score–higher weight gain until the second (5.7 cm^3^; 95% CI, 1.2-10.2 cm^3^) and third (15.3 cm^3^; 95% CI, 11.0-19.6 cm^3^) trimesters, birth (20.8 cm^3^; 95% CI, 16.4-25.1 cm^3^), 6 months (15.6 cm^3^; 95% CI, 11.2-19.9 cm^3^), 12 months (11.3 cm^3^; 95% CI, 7.0-15.6 cm^3^), and 24 months (11.1 cm^3^; 95% CI, 6.8-15.4 cm^3^) was, independent of weights at the other age windows, associated with larger total brain volume. Similarly, higher weight gain during all of the examined time periods was associated with larger subcortical gray, cerebral gray and white, and cerebellar gray and white matter volumes. After additional adjustment for childhood BMI, results remained significant (eTable 5 in the Supplement). Post hoc analyses showed that increased weight gain at birth and 6 months was associated with larger thalamus, putamen, and pallidum volumes (eTable 6 in the Supplement).

**Table 3.** Critical Periods During Fetal and Infant Life and Childhood Brain Outcomes

Fetal and infant weight SD scores	Difference (95% CI)^a^							
	Head circumference (cm)	Total brain volume (cm^3^)	Cerebral gray matter volume (cm^3^)	Cerebral white matter volume (cm^3^)	Cerebellar gray matter volume (cm^3^)	Cerebellar white matter volume (cm^3^)	Subcortical gray matter volume (cm^3^)
At 20 wk gestation	0.1 (0.0 to 0.2)^b^	5.7 (1.2 to 10.2)^c^	2.9 (0.6 to 5.1)^c^	2.2 (0.1 to 4.3)^c^	0.3 (–0.2 to 0.8)	0.1 (–0.1 to 0.2)	0.2 (0.0 to 0.4)^c^
At 30 wk gestation	0.3 (0.2 to 0.3)^b^	15.3 (11.0 to 19.6)^b^	7.3 (5.1 to 9.4)^b^	6.1 (4.1 to 8.1)^b^	1.0 (0.5 to 1.5)^b^	0.4 (0.2 to 0.5)^b^	0.5 (0.3 to 0.7)^b^
At birth	0.4 (0.3 to 0.4)^b^	20.8 (16.4 to 25.1)^b^	9.5 (7.3 to 11.7)^b^	8.7 (6.6 to 10.7)^b^	1.3 (0.8 to 1.8)^b^	0.4 (0.3 to 0.6)^b^	0.8 (0.6 to 1.0)^b^
At age 6 mo	0.4 (0.3 to 0.4)^b^	15.6 (11.2 to 19.9)^b^	6.8 (4.7 to 9.0)^b^	6.1 (4.1 to 8.1)^b^	1.4 (1.0 to 1.9)^b^	0.4 (0.3 to 0.5)^b^	0.7 (0.5 to 0.9)^b^
At age 12 mo	0.3 (0.3 to 0.4)^b^	11.3 (7.0 to 15.6)^b^	4.2 (2.1 to 6.4)^b^	4.9 (2.8 to 7.0)^b^	1.3 (0.8 to 1.8)^b^	0.4 (0.3 to 0.6)^b^	0.4 (0.2 to 0.6)^b^
At age 24 mo	0.3 (0.2 to 0.4)^b^	11.1 (6.8 to 15.4)^b^	6.0 (3.9 to 8.1)^b^	3.7 (1.7 to 5.8)^b^	0.7 (0.2 to 1.2)^b^	0.3 (0.2 to 0.4)^b^	0.4 (0.2 to 0.6)^b^

^a^ Values are linear regression coefficients (95% CIs) from conditional analyses and reflect the change in centimeters of childhood head circumference (n = 1482) and in cubic centimeters of childhood brain structures (n = 1488) for fetal and infant weight. Models are adjusted for child sex and age at the neuroimaging assessment, family income, and maternal age at intake, ethnicity, prepregnancy body mass index, educational level, smoking, alcohol use, and folic acid use during pregnancy.

^b^ *P* < .01.

^c^ *P* < .05.

### Longitudinal Fetal and Infant Growth Patterns

Compared with children with normal fetal and infant growth, those with fetal weight deceleration followed by infant weight deceleration had the smallest total brain volume (–32.5 cm^3^; 95% CI, –53.2 to –11.9 cm^3^), whereas those with fetal weight deceleration followed by infant weight acceleration had similar brain volumes. The largest brain volumes were observed for children who had both fetal and infant growth acceleration (Table 4). After additional adjustment for childhood BMI, results remained significant (eTable 7 in the Supplement). Post hoc analyses did not show significant associations for subcortical structures (eTable 8 in the Supplement).

**Table 4.** Associations of Longitudinal Fetal and Infant Growth Patterns With Childhood Brain Outcomes

Growth patterns	No.	Difference (95% CI)^a^						
		Head circumference (cm)	Total brain volume (cm^3^)	Cerebral gray matter volume (cm^3^)	Cerebral white matter volume (cm^3^)	Cerebellar gray matter volume (cm^3^)	Cerebellar white matter volume (cm^3^)	Subcortical gray matter volume (cm^3^)
Fetal growth deceleration								
Infant growth deceleration	80	–0.8 (–1.2 to –0.5)^b^	–32.5 (–53.2 to –11.9)^b^	–16.5 (–26.8 to –6.2)^b^	–10.4 (–20.0 to –0.8)^c^	–3.8 (–6.0 to –1.6)^b^	–0.8 (–1.5 to –0.2)^c^	–0.8 (–1.8 to 0.1)
Infant normal growth	245	–0.3 (–0.5 to –0.1)^c^	–8.9 (–22.2 to 4.5)	–4.4 (–11.0 to 2.3)	–2.6 (–8.8 to 3.6)	–1.1 (–2.5 to 0.3)	–0.4 (–0.8 to 0.1)	–0.4 (–1.0 to 0.2)
Infant growth acceleration	265	0.1 (–0.1 to 0.3)	–5.0 (–18.0 to 8.0)	–2.2 (–8.7 to 4.3)	–3.2 (–9.3 to 2.9)	0.5 (–0.9 to 1.9)	0.2 (–0.2 to 0.6)	–0.3 (–0.9 to 0.3)
Fetal normal growth								
Infant growth deceleration	210	–0.2 (–0.4 to 0.1)	–5.6 (–19.7 to 8.4)	–0.5 (–7.5 to 6.5)	–3.3 (–9.8 to 3.3)	–1.2 (–2.7 to 0.3)	–0.4 (–0.8 to 0.1)	–0.3 (–0.9 to 0.4)
Infant normal growth	532	[Reference]	[Reference]	[Reference]	[Reference]	[Reference]	[Reference]	[Reference]
Infant growth acceleration	274	0.5 (0.3 to 0.7)^b^	14.6 (1.7 to 27.5)^c^	6.6 (0.2 to 13.0)^c^	6.2 (0.2 to 12.2)^c^	0.7 (–0.7 to 2.1)	0.5 (0.1 to 0.8)^c^	0.7 (0.1 to 1.2)^c^
Fetal growth acceleration								
Infant growth deceleration	304	0.1 (–0.1 to 0.3)	19.2 (6.7 to 31.6)^b^	9.1 (2.9 to 15.3)^b^	7.9 (2.1 to 13.6)^b^	1.2 (–0.1 to 2.5)	0.4 (0.1 to 0.8)^c^	0.5 (–0.0 to 1.1)
Infant normal growth	313	0.5 (0.3 to 0.7)^b^	30.6 (18.3 to 42.9)^b^	15.2 (9.1 to 21.4)^b^	12.3 (6.5 to 18.0)^b^	1.2 (–0.1 to 2.6)^c^	0.6 (0.3 to 1.0)^b^	1.2 (0.6 to 1.7)^b^
Infant growth acceleration	94	1.3 (0.9 to 1.6)^b^	44.7 (25.3 to 64.1)^b^	19.8 (10.2 to 29.5)^b^	20.9 (11.9 to 29.9)^b^	1.3 (–0.8 to 3.4)	0.9 (0.3 to 1.5)^b^	1.7 (0.8 to 2.6)^b^

^a^ Values are linear regression coefficients (95% CIs) and reflect the difference in centimeters of childhood head circumference and in cubic centimeters for each childhood brain structure compared with children with normal fetal and infant growth. Models are adjusted for child sex and age at the neuroimaging assessment, family income, and maternal age at intake, ethnicity, prepregnancy body mass index, educational level, smoking, alcohol use, and folic acid use during pregnancy.

^b^ *P* < .01.

^c^ *P* < .05.

### Infant Growth

Table 5 shows that higher peak weight velocity and BMI at adiposity peak were associated with larger total brain volume (7.5 cm^3^; 95% CI, 5.6-9.3 cm^3 ^and 19.3 cm^3^; 95% CI, 14.6-23.9 cm^3^, respectively), whereas age at adiposity peak was not. Similar results were observed for subcortical gray, cerebral gray and white, and cerebellar gray and white matter volumes. Results remained significant after additional adjustment for childhood BMI (eTable 9 in the Supplement). Post hoc analyses showed that higher BMI at adiposity peak was associated with larger putamen volume (eTable 10 in the Supplement). All basic models are presented in eTables 11 through 14 in the Supplement.

**Table 5.** Associations of Infant Growth Patterns With Childhood Brain Outcomes

Characteristic	No.	Difference (95% CI)^a^						
		Head circumference (cm)	Total brain volume (cm^3^)	Cerebral gray matter volume (cm^3^)	Cerebral white matter volume (cm^3^)	Cerebellar gray matter volume (cm^3^)	Cerebellar white matter volume (cm^3^)	Subcortical gray matter volume (cm^3^)
Peak weight velocity, kg/y	2654	0.2 (0.2 to 0.2)^b^	7.5 (5.6 to 9.3)^b^	3.0 (2.1 to 3.9)^b^	3.2 (2.3 to 4.0)^b^	0.7 (0.5 to 0.9)^b^	0.2 (0.2 to 0.3)^b^	0.3 (0.2 to 0.4)^b^
BMI at adiposity peak, kg/m^2^	2489	0.5 (0.4 to 0.6)^b^	19.3 (14.6 to 23.9)^b^	8.7 (6.3 to 11.0)^b^	7.5 (5.4 to 9.7)^b^	1.5 (1.0 to 2.0)^b^	0.6 (0.5 to 0.7)^b^	0.8 (0.6 to 1.1)^b^
Age at adiposity peak, mo	2489	0.1 (0.0 to 0.2)^c^	1.5 (–5.4 to 8.4)	0.8 (–2.6 to 4.2)	0.9 (–2.3 to 4.1)	–0.1 (–0.8 to 0.7)	–0.0 (–0.2 to 0.2)	–0.1 (–0.5 to 0.2)

Abbreviation: BMI, body mass index (calculated as weight in kilograms divided by height in meters squared).

^a^ Values are linear regression coefficients (95% CIs) and reflect the change in centimeters of childhood head circumference and in cubic centimeters of childhood brain structures for peak weight velocity, BMI, and age at adiposity peak. Models are adjusted for child sex and age at the neuroimaging assessment, family income, and maternal age at intake, ethnicity, prepregnancy BMI, educational level, smoking, alcohol use, and folic acid use during pregnancy.

^b^ *P* < .01.

^c^ *P* < .05.

## Discussion

In this population-based prospective cohort study, higher gestational age and birth weight were associated with larger brain volume at 10 years of age, suggesting that both fetal life and infancy seem to be independent critical periods for childhood neurodevelopment. Our findings indicate that compared with children with normal fetal and infant growth, those with fetal weight deceleration followed by infant catch-up growth had similar brain volume at 10 years of age.

### Interpretation of Main Findings

The time from conception through 2 years of age has been recognized as a critical period of development.^1,19^ Poor growth, both in utero and in infancy, seems to influence the risk of adverse neurodevelopment outcomes later in life.^2,14,15,16,17^

Gestational age and weight at birth have been largely used as determinants of child health. Previous studies have focused on infants born preterm or with low birth weight and have shown overall and regional reductions in brain volumes during childhood and adolescence.^10,11,12^ Recent results from our own research group suggest an association of higher gestational age at birth, even within the term range, with larger brain volumes in 10-year-old children.^13^ Our current findings show positive associations of gestational age, weight, and size for gestational age at birth with childhood head circumference and total, cerebral gray and white, and cerebellar gray and white matter volumes. Previous studies showed that birth weight in the upper growth centiles is also associated with both short- and long-term adverse neurodevelopment outcomes.^48,49^ We could not detect nonlinear associations in our study. Further studies in clinical populations are needed to identify the specific optimal ranges for fetal and infant growth.

As fetal and infant growth are correlated with each other, it is important to study their independent associations with childhood brain morphology. We observed that faster weight gain during each of the studied periods between midgestation and 24 months of age was associated with larger brain volumes independent of growth during any other age windows. The largest differences in brain volume were observed in relation to weight gain from late gestation to birth, whereas effect estimates were smallest in magnitude for growth after 6 months of age. This finding is in line with previous evidence that late gestation and the first postnatal months represent a critical period of brain development, during which the human brain experiences a striking growth spurt.^50^

To the best of our knowledge, the current study is the first to examine the associations of prospectively assessed fetal and infant growth patterns with childhood brain morphology measured by MRI. As compared with children with normal fetal and infant growth, those who experienced fetal weight deceleration but showed postnatal catch-up growth had similar brain volumes at 10 years. In contrast, a previous cross-sectional study in the Netherlands comparing brain morphology in children born SGA (n = 36) with those born AGA (n = 19) suggested that being born SGA, even after postnatal catch-up growth, was associated with reduced cerebral and cerebellar white matter volumes at the age of 4 to 7 years.^51^ We also observed that higher peak weight velocity and BMI at adiposity peak were, independent of childhood BMI, associated with larger brain volumes. A previous study among 613 infants born preterm reported that greater infant weight and BMI gain to term were associated with better cognitive functioning.^24^ A Finnish cohort of children born very preterm and with very low birth weight found that faster infant weight gain and head growth were associated with higher IQ among AGA children.^23^ Altogether, these findings suggest that faster growth during the first 2 years of life might be associated with improved neurodevelopment outcomes later in life. As the current study focused on brain volumes only, caution is warranted regarding potential functional implications of the observed associations.

Fetal programming mechanisms might partly explain these associations. Maternal age, substance use, poor diet, and psychological distress are known to influence fetal growth. An adverse intrauterine environment may lead to adaptive fetal responses, eg, circulatory redistribution, and negatively affect brain development.^52^ Such stress-related factors may persist after pregnancy and alter infant brain growth. The current study suggests that suboptimal fetal or infant growth may be associated with fetal programming differences in brain outcomes in the longer term.^53^ Another potential mechanism is genetic predisposition.^54^ Previous studies have found that the genetic background of birth weight is also associated with head circumference at birth and in later life.^55,56^

Altogether, the findings of the current study suggest that fetal and infant weight growth are associated with brain morphology in school-age children. Even though the observed associations are relatively small, they are important from a developmental and preventive perspective. Early postnatal life seems to be a window of opportunity to improve childhood growth and evaluate the healthy consequences for brain development. Future research should investigate potential causal pathways and underlying mechanisms of the observed associations and explore whether these associations link to neurocognitive outcomes.

### Limitations

This study has some limitations. Because mothers of children with and without good-quality brain MRI data available were different regarding socioeconomic and lifestyle characteristics, we cannot exclude the possibility of selection bias. We had a relatively healthy population. Children born preterm or SGA do not reflect the full neonatal group, which mainly comprised children born late preterm after 35 weeks or children with mild SGA. Also, fetal weight estimated by ultrasound might be prone to measurement error, mainly among extremes (ie, fetuses with low or high weight), which might have influenced our findings.^57^ Further, for the infant weight change analysis, we considered the period from birth to 24 months. When weight was missing at 24 months, we used weight at 12 or 6 months. We used all data available to optimize statistical power but might have introduced bias. Brain morphology has been associated with behavioral and cognitive functions.^58^ Future studies should investigate repeated assessments of brain morphology as well as multimodal neuroimaging in combination with behavioral and cognitive functioning in large population-based cohorts. Finally, owing to the observational design of the study, unmeasured residual confounding might still be present.

## Conclusions

The results of this cohort study suggest that early-life growth is associated with brain morphology later in childhood. Both fetal life and infancy seem to be important critical periods of childhood brain development. Also, faster infant growth in the first 2 years of life may be associated with improved brain development later in life. Further studies are needed to assess whether a causal relationship exists and focus on potential long-term consequences of early growth patterns on brain structure and function.

## Notes

Supplement. **eFigure 1.** Flowchart of study population **eFigure 2.** Directed acyclic graph showing the hypothesized relationship between childhood fetal and infant growth and brain outcomes and the covariates **eTable 1.** Fetal and infant growth measures **eTable 2.** Comparison of characteristics between responders and non-responders **eTable 3.** Associations of birth outcomes with childhood brain outcomes (BMI models) **eTable 4.** Associations of birth outcomes with childhood brain outcomes (subcortical structures) **eTable 5.** Critical periods during fetal and infant life and childhood brain outcomes (BMI models) **eTable 6.** Critical periods during fetal and infant life and childhood brain outcomes (subcortical structures) **eTable 7.** Associations of longitudinal fetal and infant growth patterns with childhood brain outcomes (BMI models) **eTable 8.** Associations of longitudinal fetal and infant growth patterns with childhood brain outcomes (subcortical structures) **eTable 9.** Associations of infant growth patterns with childhood brain outcomes (BMI models) **eTable 10.** Associations of infant growth patterns with childhood brain outcomes (subcortical structures) **eTable 11.** Associations of birth outcomes with childhood brain outcomes (basic models) **eTable 12.** Critical periods during fetal and infant life and childhood brain outcomes (basic models) **eTable 13.** Associations of longitudinal fetal and infant growth patterns with childhood brain outcomes (basic models) **eTable 14.** Associations of infant growth patterns with childhood brain outcomes (basic models)
