# Supplementary material for: Patterns of Fetal and Infant Growth and Brain Morphology at Age 10 Years
Source: JAMA Netw Open. 2021 Dec 9;4(12):e2138214. doi: 10.1001/jamanetworkopen.2021.38214 (PMC8662367; doi:10.1001/jamanetworkopen.2021.38214)
Supplement: Supplement. — eFigure 1. Flowchart of study population eFigure 2. Directed acyclic graph showing the hypothesized relationship between childhood fetal and infant growth and brain outcomes and the covariates eTable 1. Fetal and infant growth measures eTable 2. Comparison of characteristics between responders and non-responders eTable 3. Associations of birth outcomes with childhood brain outcomes (BMI models) eTable 4. Associations of birth outcomes with childhood brain outcomes (subcortical structures) eTable 5. Critical periods during fetal and infant life and childhood brain outcomes (BMI models) eTable 6. Critical periods during fetal and infant life and childhood brain outcomes (subcortical structures) eTable 7. Associations of longitudinal fetal and infant growth patterns with childhood brain outcomes (BMI models) eTable 8. Associations of longitudinal fetal and infant growth patterns with childhood brain outcomes (subcortical structures) eTable 9. Associations of infant growth patterns with childhood brain outcomes (BMI models) eTable 10. Associations of infant growth patterns with childhood brain outcomes (subcortical structures) eTable 11. Associations of birth outcomes with childhood brain outcomes (basic models) eTable 12. Critical periods during fetal and infant life and childhood brain outcomes (basic models) eTable 13. Associations of longitudinal fetal and infant growth patterns with childhood brain outcomes (basic models) eTable 14. Associations of infant growth patterns with childhood brain outcomes (basic models) [file jamanetwopen-e2138214-s001.pdf]

Supplemental Online Content

Silva CCV, El Marroun H, Sammallahti S, et al. Patterns of fetal and infant growth and brain morphology at age 10 years. *JAMA Netw Open.* 2021;4(12):e2138214. doi:10.1001/jamanetworkopen.2021.38214

eFigure 1. Flowchart of study population

eFigure 2. Directed acyclic graph showing the hypothesized relationship between childhood fetal and infant growth and brain outcomes and the covariates

eTable 1. Fetal and infant growth measures

eTable 2. Comparison of characteristics between responders and non-responders

eTable 3. Associations of birth outcomes with childhood brain outcomes (BMI models)

eTable 4. Associations of birth outcomes with childhood brain outcomes (subcortical structures)

eTable 5. Critical periods during fetal and infant life and childhood brain outcomes (BMI models)

eTable 6. Critical periods during fetal and infant life and childhood brain outcomes (subcortical structures)

eTable 7. Associations of longitudinal fetal and infant growth patterns with childhood brain outcomes (BMI models)

eTable 8. Associations of longitudinal fetal and infant growth patterns with childhood brain outcomes (subcortical structures)

eTable 9. Associations of infant growth patterns with childhood brain outcomes (BMI models)

eTable 10. Associations of infant growth patterns with childhood brain outcomes (subcortical structures)

eTable 11. Associations of birth outcomes with childhood brain outcomes (basic models)

eTable 12. Critical periods during fetal and infant life and childhood brain outcomes (basic models)

eTable 13. Associations of longitudinal fetal and infant growth patterns with childhood brain outcomes (basic models)

eTable 14. Associations of infant growth patterns with childhood brain outcomes (basic models)

This supplemental material has been provided by the authors to give readers additional information about their work.

© 2021 Silva CCV et al. *JAMA Network Open.*

eFigure 1. Flowchart of study population.

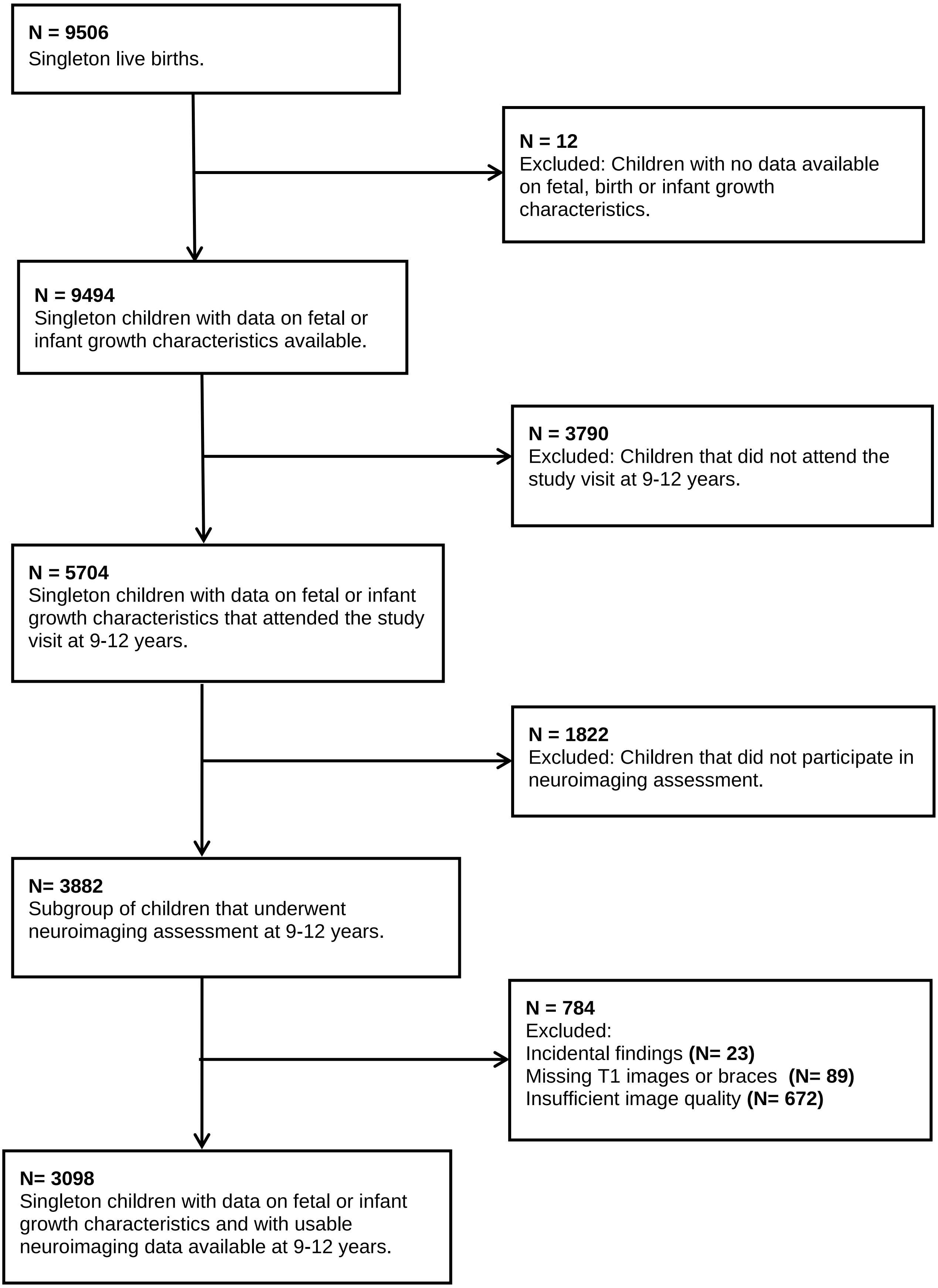

© 2021 Silva CCV et al. *JAMA Network Open.*

eFigure 2. Directed acyclic graph showing the hypothesized relationship between childhood fetal and infant growth and brain outcomes and the covariates

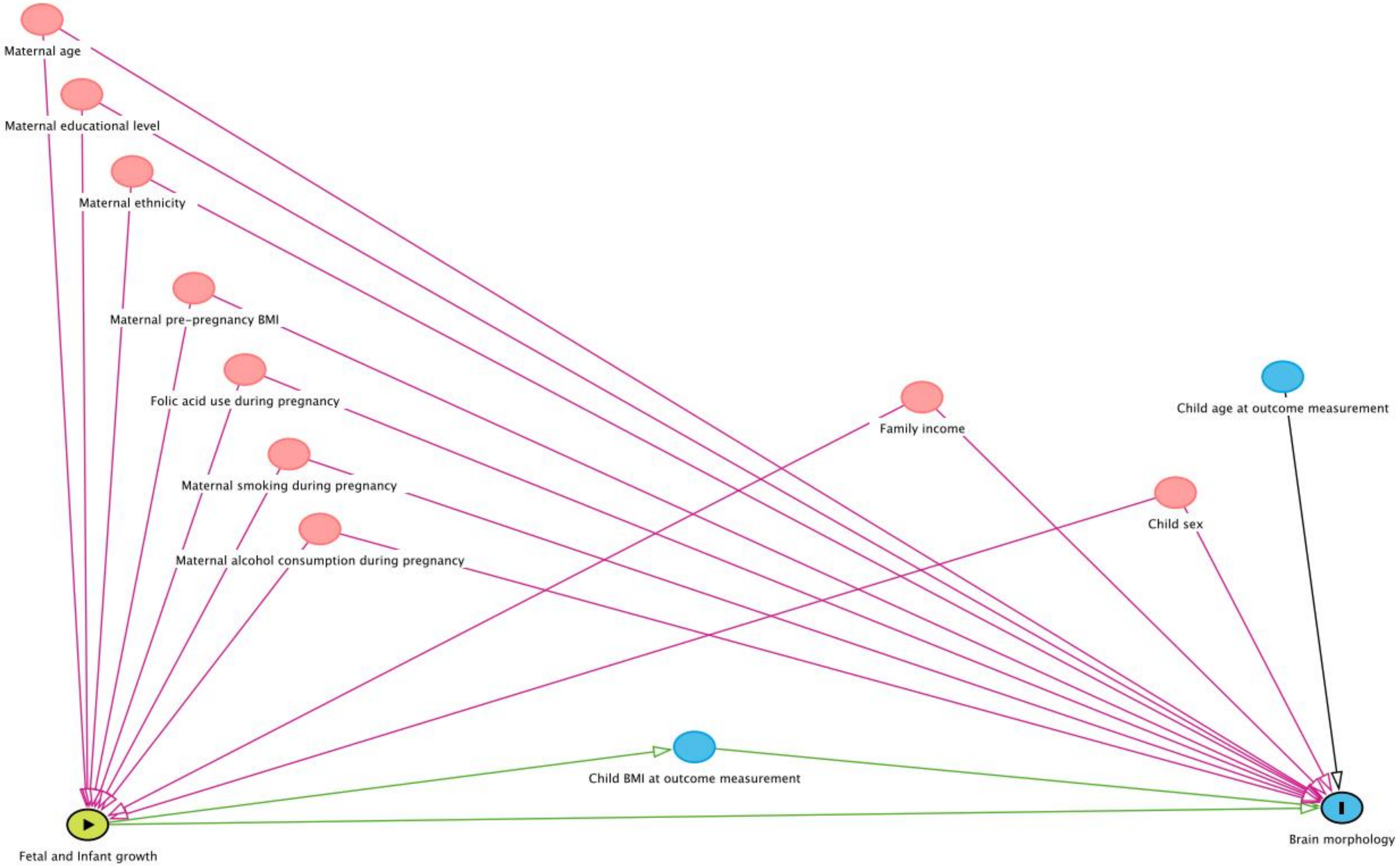

© 2021 Silva CCV et al. *JAMA Network Open.*

eTable 1. Fetal and infant growth measures

Fetal and Infant growth	Age of assessment	Technique/information source	Outcome/measure	References
**Fetal growth measures**				
Gestational age	1st trimester	Ultrasound.	Continuous and categorized into preterm (<37 weeks), term (37-42 weeks) or post-term (>42 weeks).	Tunon K, et al. A comparison between ultrasound and a reliable last menstrual period as predictors of the day of delivery in 15,000 examinations. Ultrasound Obstet Gynecol. 1996;8(3):178-85.
Head circumference	2nd and 3rd trimester	Ultrasound.	Used to estimate fetal weight.	Kooijman MN, et al. The Generation R Study: design and cohort update 2017. Eur J Epidemiol. 2016 ;31(12):1243-64.
Abdominal circumference				
Femur length				
Estimated fetal weight	2nd and 3rd trimester	Calculated using the Hadlock formula.	Gestational-age-adjusted SDS for weight calculated using reference growth curves derived from the same cohort as the current study.	Hadlock FP,et al. Estimation of fetal weight with the use of head, body, and femur measurements--a prospective study. Am J Obstet Gynecol. 1985;151(3):333-7.<br>Verburg BO,et al. New charts for ultrasound dating of pregnancy and assessment of fetal growth: longitudinal data from a population-based cohort study. Ultrasound Obstet Gynecol. 2008;31(4):388-96.
**Infant growth measures**				
Weight	Birth	Medical records.	Sex- and gestational age-adjusted SDS for weight calculated based on North European reference charts.	Niklasson A, et al. An update of the Swedish reference standards for weight, length and head circumference at birth for given gestational age (1977-1981). Acta Paediatr Scand. 1991;80(8-9):756-62.
	6, 12 and 24 months	Community health centers.	Sex- and age-adjusted SDS for weight calculated based on Dutch reference growth charts.	Fredriks AM, et al. Continuing positive secular growth change in The

© 2021 Silva CCV et al. *JAMA Network Open.*

				Netherlands 1955-1997. Pediatr Res. 2000;47(3):316-23.
Peak weight velocity	From birth to 36 months	Derived using the Reed1 model by sex on all weight measures.	Reflects the greatest weight growth in infancy.	Berkey CS, Reed RB. A model for describing normal and abnormal growth in early childhood. Hum Biol. 1987;59(6):973-87.
BMI at adiposity peak	From 14 days to 18 months	Cubic mixed-effects model was fitted on log (BMI), adjusted for sex.	BMI was derived for each individual and the point where the curve reaches its maximum gives BMI and age at adiposity peak.	Sovio U, et al. Genetic determinants of height growth assessed longitudinally from infancy to adulthood in the northern Finland birth cohort 1966. PLoS Genet. 2009;5(3):e1000409.
Age at adiposity peak				
**Fetal and Infant growth patterns**				
Growth deceleration, normal growth or growth acceleration	From 2nd trimester to birth	Fetal weight change was the growth between the 2nd trimester and birth. Infant weight change was the growth from birth to 24 months. Growth acceleration or deceleration were defined as a change greater than 0.67 SD between time points.	Fetal and infant weight change were categorized into 3 groups (growth deceleration, normal growth, and growth acceleration), and combined variables that reflect 9 different growth patterns were created.	Ong KK, et al. Association between postnatal catch-up growth and obesity in childhood: prospective cohort study. BMJ. 2000;320(7240):967-71.

© 2021 Silva CCV et al. *JAMA Network Open.*

eTable 2. Comparison of characteristics between responders and non-responders[a]

Characteristics	Responders (n= 3098)	Non-Responders (n= 2606)	P Value[b]
**Maternal characteristics**			
Age at intake, mean (SD), years	31.1 (4.9)	30.8 (5.1)	< 0.05
Ethnicity, N(%)	3032	2521	< 0.05
Dutch	1753 (57.8)	1422 (56.4)	
Non-Dutch, Western	253 (8.3)	204 (8.1)	
Non-Dutch, Non-Western	1026 (33.8)	895 (35.5)	
Prepregnancy body mass index, median (95% range), kg/m2	22.5 (18.0-34.8)	22.7 (18.2-34.3)	0.10
Education, N(%)	2848	2389	< 0.001
Primary school	186 (6.5)	220 (9.2)	
Secondary school	1156 (40.6)	1051 (44.0)	
Higher education	1506 (52.9)	1118 (46.8)	
Monthly household income, US$	2409	2009	< 0.001
< 1200	330 (13.7)	324 (16.1)	
1200-2000	380 (15.8)	381 (19.0)	
> 2000	1699 (70.5)	1304 (64.9)	
Folic acid used, N(%)	2173	1790	< 0.001
Yes	1737 (79.9)	1334 (74.5)	
Alcohol consumption, N (%)	2449	2086	< 0.05
Yes	1238 (50.6)	992 (47.6)	
Smoking, N (%)	2450	2074	
Yes	501 (20.4)	477 (23.0)	< 0.05
**Fetal characteristics**			
Second trimester, median (95% range)			
Gestational age, weeks	20.5 (18.7-23.3)	20.6 (18.5-23.5)	< 0.05
Estimated fetal weight, g	362 (248-612)	367 (244-640)	< 0.05
Third trimester, median (95% range)			
Gestational age, weeks	30.4 (28.5-32.7)	30.4 (28.4-33.2)	0.11
Estimated fetal weight, g	1602 (1186-2150)	1601 (1171-2295)	0.20
**Birth characteristics**			
Gestational age at birth, median (95% range), weeks	40.1 (36.0-42.3)	40.1 (35.6-42.3)	0.05
Sex, N (%)			0.89
Girls	1557 (50.3)	1305 (50.1)	
Birth weight, mean (SD), g	3446 (553)	3440 (554)	0.06
**Infant characteristics**			
At 6 months, median (95% range)			
Age at visit, months	6.2 (5.2-7.9)	6.2 (5.3-8.9)	0.35
Weight, kg	7.8 (6.2-9.7)	7.8 (6.2-9.8)	0.35
At 12 months, median (95% range)			
Age at visit, months	11.0 (10.1-13.0)	11.1 (10.2-12.5)	< 0.001
Weight, kg	9.6 (7.7-11.8)	9.6 (7.6-12.0)	0.77
At 24 months, median (95% range)			
Age at visit, months	24.8 (23.4-28.1)	24.9 (23.4-28.3)	< 0.05
Weight, kg	12.8 (10.2-16.1)	12.9 (10.3-16.1)	0.55
Peak weight velocity, mean (SD), kg/y	12.1 (2.1)	12.2 (2.1)	0.62
Body mass index at adiposity peak, mean (SD), kg/m^2	17.6 (0.8)	17.6 (0.8)	0.48
Age at adiposity peak, median (95% range), months	8.4 (7.8-9.6)	8.4 (7.8-9.6)	0.14
**Child characteristics**			
Age at MRI, mean (SD), years	10.1 (0.6)	10.2 (0.8)	< 0.05
Length, mean (SD), cm	141.6 (6.6)	141.5 (6.8)	0.43
Weight, median (95% range), kg	33.8 (24.4-53.0)	34.0 (26.4-51.0)	0.37
Body mass index, median (95% range), kg/m^2	16.9 (14.1-24.3)	17.0 (14.3-23.5)	0.10

[a] Values are means (standard deviation), medians (95% range) or numbers of subjects (valid %).
[b] P-values for differences in subject characteristics between groups were calculated performing independent sample t-tests for normally distributed continuous variables, Mann-Whitney test for not normally distributed continuous variables and chi-square tests for categorical variables.

© 2021 Silva CCV et al. *JAMA Network Open.*

eTable 3. Associations of birth outcomes with childhood brain outcomes (BMI models)

	Difference (95% Confidence Interval)						
**Birth outcomes**	**Head circumference (cm)**	**Total brain volume (cm^3)**	**Cerebral gray matter volume (cm^3)**	**Cerebral white matter volume (cm^3)**	**Cerebellar gray matter volume (cm^3)**	**Cerebellar white matter volume (cm^3)**	**Subcortical gray matter volume (cm^3)**
**Gestational age, week**	0.1 (0.0 to 0.1)**	4.8 (3.0 to 6.6)**	2.7 (1.8 to 3.6)**	1.3 (0.4 to 2.1)**	0.4 (0.2 to 0.6)**	0.1 (0.1 to 0.2)**	0.3 (0.2 to 0.4)**
< 37 weeks (N=138)	-0.2 (-0.5 to 0.0)	-29.0 (-44.5 to -13.4)**	-17.1 (-24.8 to -9.4)**	-6.6 (-13.8 to 0.5)	-2.8 (-4.5 to -1.2)**	-0.8 (-1.2 to -0.3)**	-1.5 (-2.2 to -0.8)**
37-41 weeks (N=2718)	[Reference]	[Reference]	[Reference]	[Reference]	[Reference]	[Reference]	[Reference]
>42 weeks (N=223)	0.3 (0.1 to 0.5)**	11.5 (-1.0 to 24.0)	7.4 (1.2 to 13.5)*	2.0 (-3.8 to 7.7)	0.8 (-0.5 to 2.2)	0.4 (-0.0 to 0.7)	1.0 (0.4 to 1.5)**
**Birth weight, 500g**	0.3 (0.2 to 0.3)**	20.0 (17.0 to 22.9)**	9.7 (8.3 to 11.2)**	7.6 (6.2 to 9.0)**	1.4 (1.1 to 1.7)**	0.4 (0.3 to 0.5)**	0.8 (0.7 to 1.0)**
<2500 g (N=126)	-0.6 (-0.9 to -0.3)**	-44.3 (-60.5 to -28.1)**	-20.9 (-28.9 to -12.9)**	-16.1 (-23.5 to -8.6)**	-3.6 (-5.3 to -1.9)**	-1.5 (-2.0 to -1.0)**	-2.1 (-2.9 to -1.4)**
2500-4500 g (N=2890)	[Reference]	[Reference]	[Reference]	[Reference]	[Reference]	[Reference]	[Reference]
>4500 g (N=78)	0.6 (0.3 to 0.9)**	38.5 (18.1 to 58.9)**	18.3 (8.1 to 28.4)**	16.8 (7.4 to 26.2)**	1.5 (-0.7 to 3.7)	0.4 (-0.2 to 1.0)	1.5 (0.6 to 2.4)**
**Size for gestational age, SDS**	0.3 (0.2 to 0.3)**	21.5 (18.3 to 24.7)**	10.0 (8.4 to 11.6)**	8.9 (7.4 to 10.4)**	1.4 (1.1 to 1.8)**	0.4 (0.3 to 0.5)**	0.8 (0.6 to 0.9)**
Small (<10 percentile) (N=307)	-0.6 (-0.7 to -0.4)**	-36.1 (-46.8 to -25.3)**	-16.6 (-21.9 to -11.2)**	-15.2 (-20.1 to -10.2)**	-2.3 (-3.5 to -1.2)**	-0.7 (-1.0 to -0.4)**	-1.3 (-1.8 to -0.8)**
Appropriate (10-90 percentile) (N=2458)	[Reference]	[Reference]	[Reference]	[Reference]	[Reference]	[Reference]	[Reference]
Large (>90 percentile) (N=307)	0.5 (0.3 to 0.6)**	33.0 (22.2 to 43.8)**	14.2 (8.8 to 19.5)**	14.8 (9.8 to 19.8)**	2.2 (1.0 to 3.4)**	0.6 (0.2 to 0.9)**	1.3 (0.8 to 1.8)**

Values are linear regression coefficients (95% confidence intervals) and reflect the change in cm of childhood head circumference and in cm^3 of childhood brain structures for birth outcomes. Models are adjusted for child sex, BMI and age at the neuroimaging assessment, family income and maternal age at intake, ethnicity, pre-pregnancy BMI, educational level, smoking, alcohol, and folic acid use during pregnancy. *$p < 0.05$. ** $p < 0.01$.

© 2021 Silva CCV et al. *JAMA Network Open.*

eTable 4. Associations of birth outcomes with childhood brain outcomes (subcortical structures)

	Difference (95% Confidence Interval)						
**Birth outcomes**	**Thalamus volume (cm^3)**	**Amygdala volume (cm^3)**	**Hippocampus volume (cm^3)**	**Globus Pallidus volume (cm^3)**	**Putamen volume (cm^3)**	**Caudate nucleus volume (cm^3)**	**Nucleus accumbens volume (cm^3)**
**Gestational age, week**	0.07 (0.05 to 0.08)**	0.00 (-0.01 to 0.01)	0.01 (-0.01 to 0.02)	0.02 (0.01 to 0.03)**	0.03 (0.01 to 0.05)**	0.02 (0.00 to 0.03)*	0.00 (-0.00 to 0.01)
**Birth weight, 500g**	0.09 (0.06 to 0.12)**	-0.01 (-0.02 to -0.00)*	-0.00 (-0.03 to 0.01)	0.03 (0.02 to 0.04)**	0.05 (0.01 to 0.08)**	0.02 (-0.00 to 0.05)	0.01 (00.0 to 0.02)**
**Size for gestational age, SDS**	0.03 (-0.00 to 0.06)	-0.02 (-0.03 to -0.01)**	-0.02 (-0.04 to 0.00)	0.02 (0.01 to 0.03)**	0.02 (-0.02 to 0.06)	0.01 (-0.02 to 0.04)	0.01 (0.00 to 0.02)**

Values are linear regression coefficients (95% confidence intervals) and reflect the change in cm of childhood head circumference and in cm^3 of childhood brain structures for birth outcomes. Models are adjusted for child sex, intracranial volume and age at the neuroimaging assessment, family income and maternal age at intake, ethnicity, pre-pregnancy BMI, educational level, smoking, alcohol, and folic acid use during pregnancy. *p < 0.05. ** p < 0.01.

© 2021 Silva CCV et al. *JAMA Network Open.*

eTable 5. Critical periods during fetal and infant life and childhood brain outcomes (BMI models)

	Difference (95% Confidence Interval)						
**Fetal and Infant Weight Standard Deviation Scores**	**Head circumference (cm)**	**Total brain volume (cm^3)**	**Cerebral gray matter volume (cm^3)**	**Cerebral white matter volume (cm^3)**	**Cerebellar gray matter volume (cm^3)**	**Cerebellar white matter volume (cm^3)**	**Subcortical gray matter volume (cm^3)**
At 20 weeks	0.1 (0.0 to 0.2)**	5.6 (1.1 to 10.1)*	2.9 (0.7 to 5.2)*	2.1 (0.0 to 4.2)	0.4 (-0.1 to 0.9)	0.1 (-0.1 to 0.2)	0.2 (-0.0 to 0.4)
At 30 weeks	0.2 (0.2 to 0.3)**	15.9 (11.6 to 20.2)**	7.6 (5.5 to 9.8)**	6.3 (4.3 to 8.4)**	1.1 (0.6 to 1.5)**	0.3 (0.2 to 0.5)**	0.5 (0.3 to 0.7)**
At birth	0.3 (0.3 to 0.4)**	21.1 (16.8 to 25.5)**	9.7 (7.6 to 11.9)**	8.8 (6.7 to 10.9)**	1.4 (0.9 to 1.9)**	0.4 (0.2 to 0.5)**	0.8 (0.6 to 1.0)**
At 6 months	0.3 (0.2 to 0.3)**	16.4 (11.9 to 20.8)**	7.3 (5.1 to 9.6)**	6.4 (4.3 to 8.5)**	1.6 (1.1 to 2.1)**	0.3 (0.1 to 0.4)**	0.7 (0.5 to 0.9)**
At 12 months	0.3 (0.2 to 0.3)**	12.3 (7.9 to 16.7)**	4.8 (2.6 to 7.0)**	5.3 (3.2 to 7.4)**	1.4 (1.0 to 1.9)**	0.3 (0.2 to 0.5)**	0.4 (0.2 to 0.6)**
At 24 months	0.2 (0.1 to 0.3)**	11.8 (7.4 to 16.2)**	6.4 (4.2 to 8.6)**	4.0 (2.0 to 6.1)**	0.8 (0.3 to 1.3)**	0.2 (0.0 to 0.3)*	0.4 (0.2 to 0.6)**

Values are linear regression coefficients (95% confidence intervals) from conditional analyses and reflect the change in cm of childhood head circumference (N=1482) and in cm^3 of childhood brain structures (N=1488) for fetal and infant weight. Models are adjusted for child sex, BMI and age at the neuroimaging assessment, family income and maternal age at intake, ethnicity, pre-pregnancy BMI, educational level, smoking, alcohol, and folic acid use during pregnancy. *p < 0.05. ** p < 0.01.

© 2021 Silva CCV et al. *JAMA Network Open.*

eTable 6. Critical periods during fetal and infant life and childhood brain outcomes (subcortical structures)

	Difference (95% Confidence Interval)						
**Fetal and Infant Weight Standard Deviation Scores**	**Thalamus volume (cm^3)**	**Amygdala volume (cm^3)**	**Hippocampus volume (cm^3)**	**Globus Pallidus volume (cm^3)**	**Putamen volume (cm^3)**	**Caudate nucleus volume (cm^3)**	**Nucleus accumbens volume (cm^3)**
At 20 weeks	0.01 (-0.04 to 0.05)	0.00 (-0.01 to 0.02)	0.00 (-0.03 to 0.03)	-0.01 (-0.03 to 0.01)	-0.02 (-0.07 to 0.03)	0.00 (-0.04 to 0.04)	0.00 (-0.01 to 0.01)
At 30 weeks	0.02 (-0.03 to 0.06)	-0.01 (-0.03 to 0.00)	0.01 (-0.02 to 0.04)	0.01 (-0.01 to 0.03)	0.01 (-0.04 to 0.06)	-0.01 (-0.05 to 0.03)	0.01 (0.00 to 0.02)*
At birth	0.08 (0.04 to 0.13)**	-0.01 (-0.03 to 0.00)	-0.00 (-0.03 to 0.03)	0.03 (0.01 to 0.04)**	0.04 (-0.01 to 0.09)	0.01 (-0.03 to 0.05)	0.01 (00.0 to 0.02)*
At 6 months	0.05 (0.01 to 0.09)*	0.01 (-0.00 to 0.03)	0.02 (-0.01 to 0.05)	0.02 (0.00 to 0.04)*	0.08 (0.03 to 0.13)**	0.03 (-0.02 to 0.07)	0.00 (-0.01 to 0.01)
At 12 months	-0.00 (-0.05 to 0.04)	0.01 (-0.01 to 0.02)	0.03 (-0.00 to 0.06)	-0.01 (-0.03 to 0.01)	-0.03 (-0.08 to 0.02)	0.01 (-0.03 to 0.05)	-0.00 (-0.01 to 0.01)
At 24 months	-0.01 (-0.06 to 0.03)	-0.01 (-0.02 to 0.01)	0.02 (-0.01 to 0.05)	-0.01 (-0.03 to 0.01)	-0.01 (-0.05 to 0.04)	-0.03 (-0.07 to 0.02)	-0.01 (-0.01 to 0.00)

Values are linear regression coefficients (95% confidence intervals) from conditional analyses and reflect the change in cm of childhood head circumference (N=1482) and in cm^3 of childhood brain structures (N=1488) for fetal and infant weight. Models are adjusted for child sex, intracranial volume and age at the neuroimaging assessment, family income and maternal age at intake, ethnicity, pre-pregnancy BMI, educational level, smoking, alcohol, and folic acid use during pregnancy. *p < 0.05. ** p < 0.01.

© 2021 Silva CCV et al. *JAMA Network Open.*

eTable 7. Associations of longitudinal fetal and infant growth patterns with childhood brain outcomes (BMI models)

	Difference (95% Confidence Interval)						
**Growth patterns**	**Head circumference (cm)**	**Total brain volume (cm^3)**	**Cerebral gray matter volume (cm^3)**	**Cerebral white matter volume (cm^3)**	**Cerebellar gray matter volume (cm^3)**	**Cerebellar white matter volume (cm^3)**	**Subcortical gray matter volume (cm^3)**
**Fetal growth deceleration**							
Infant growth deceleration (N= 80)	-0.5 (-0.9 to -0.2)**	-28.6 (-49.4 to -7.8)**	-14.8 (-25.2 to -4.5)**	-8.9 (-18.6 to 0.8)	-3.6 (-5.9 to -1.4)**	-0.5 (-1.1 to 0.1)	-0.6 (-1.6 to 0.3)
Infant normal growth (N= 245)	-0.2 (-0.4 to 0.0)	-7.8 (-21.2 to 5.6)	-4.0 (-10.7 to 2.7)	-2.1 (-8.4 to 4.1)	-1.1 (-2.5 to 0.3)	-0.2 (-0.6 to 0.2)	-0.3 (-0.9 to 0.3)
Infant growth acceleration (N= 265)	0.0 (-0.2 to 0.2)	-6.4 (-19.5 to 6.6)	-2.8 (-9.4 to 3.7)	-3.7 (-9.8 to 2.4)	0.4 (-1.0 to 1.9)	0.0 (-0.4 to 0.4)	-0.4 (-0.9 to 0.2)
**Fetal normal growth**							
Infant growth deceleration (N= 210)	0.0 (-0.3 to 0.2)	-2.7 (-16.8 to 11.4)	0.7 (-6.3 to 7.7)	-2.1 (-8.6 to 4.5)	-1.1 (-2.6 to 0.4)	-0.2 (-0.6 to 0.3)	-0.1 (-0.7 to 0.5)
Infant normal growth (N= 532)	[Reference]	[Reference]	[Reference]	[Reference]	[Reference]	[Reference]	[Reference]
Infant growth acceleration (N= 274)	0.3 (0.1 to 0.5)**	12.7 (-0.3 to 25.7)	5.8 (-0.6 to 12.3)	5.5 (-0.5 to 11.6)	0.6 (-0.8 to 2.0)	0.2 (-0.2 to 0.6)	0.6 (-0.0 to 1.1)
**Fetal growth acceleration**							
Infant growth deceleration (N= 304)	0.2 (0.0 to 0.4)	20.7 (8.2 to 33.1)**	9.7 (3.5 to 15.9)**	8.5 (2.7 to 14.3)**	1.3 (-0.1 to 2.6)	0.5 (0.2 to 0.9)**	0.6 (0.1 to 1.2)*
Infant normal growth (N= 313)	0.4 (0.2 to 0.6)**	30.1 (17.7 to 42.5)**	15.2 (9.1 to 21.4)**	11.9 (6.1 to 17.7)**	1.4 (0.0 to 2.7)*	0.5 (0.1 to 0.8)*	1.1 (0.5 to 1.6)**
Infant growth acceleration (N= 94)	1.0 (0.7 to 1.3)**	39.9 (20.4 to 59.4)**	17.9 (8.1 to 27.6)**	18.9 (9.9 to 28.0)**	1.1 (-1.0 to 3.2)	0.5 (-0.1 to 1.1)	1.5 (0.6 to 2.3)**

Values are linear regression coefficients (95% confidence intervals) and reflect the difference in cm of childhood head circumference and in cm^3 for each childhood brain structures compared to children with normal fetal and infant growth. Models are adjusted for child sex, BMI and age at the neuroimaging assessment, family income and maternal age at intake, ethnicity, pre-pregnancy BMI, educational level, smoking, alcohol, and folic acid use during pregnancy. *p < 0.05. ** p < 0.01.

© 2021 Silva CCV et al. *JAMA Network Open.*

eTable 8. Associations of longitudinal fetal and infant growth patterns with childhood brain outcomes (subcortical structures)

	Difference (95% Confidence Interval)						
**Growth patterns**	**Thalamus volume (cm^3)**	**Amygdala volume (cm^3)**	**Hippocampus volume (cm^3)**	**Globus Pallidus volume (cm^3)**	**Putamen volume (cm^3)**	**Caudate nucleus volume (cm^3)**	**Nucleus accumbens volume (cm^3)**
**Fetal growth acceleration**							
Infant growth deceleration (N= 304)	-0.02 (-0.13 to 0.09)	-0.05 (-0.08 to -0.01)*	-0.01 (-0.08 to 0.06)	0.02 (-0.02 to 0.06)	0.08 (-0.04 to 0.19)	-0.04 (-0.13 to 0.06)	0.02 (0.00 to 0.04)*
Infant normal growth (N= 313)	0.03 (-0.08 to 0.13)	-0.00 (-0.04 to 0.03)	0.02 (-0.05 to 0.09)	0.01 (-0.03 to 0.05)	0.02 (-0.09 to 0.14)	0.05 (-0.05 to 0.14)	0.02 (-0.00 to 0.04)
Infant growth acceleration (N= 94)	-0.06 (-0.25 to 0.12)	-0.02 (-0.08 to 0.04)	0.01 (-0.11 to 0.13)	-0.04 (-0.11 to 0.03)	0.06 (-0.14 to 0.26)	-0.09 (-0.26 to 0.07)	-0.01 (-0.05 to 0.02)

Values are linear regression coefficients (95% confidence intervals) and reflect the difference in cm of childhood head circumference and in cm^3 for each childhood brain structures compared to children with normal fetal and infant growth. Models are adjusted for child sex, intracranial volume and age at the neuroimaging assessment, family income and maternal age at intake, ethnicity, pre-pregnancy BMI, educational level, smoking, alcohol, and folic acid use during pregnancy. *p < 0.05. ** p < 0.01.

© 2021 Silva CCV et al. *JAMA Network Open.*

eTable 9. Associations of infant growth patterns with childhood brain outcomes (BMI models)

	Difference (95% Confidence Interval)						
**Characteristic**	**Head circumference (cm)**	**Total brain volume (cm^3)**	**Cerebral gray matter volume (cm^3)**	**Cerebral white matter volume (cm^3)**	**Cerebellar gray matter volume (cm^3)**	**Cerebellar white matter volume (cm^3)**	**Subcortical gray matter volume (cm^3)**
Peak weight velocity, kg/y (N=2654)	0.1 (0.1 to 0.2)**	6.7 (4.7 to 8.6)**	2.7 (1.7 to 3.6)**	2.9 (2.0 to 3.7)**	0.7 (0.5 to 0.9)**	0.1 (0.1 to 0.2)**	0.3 (0.2 to 0.3)**
BMI at adiposity peak, kg/m^2 (N=2489)	0.3 (0.2 to 0.4)**	17.7 (12.7 to 22.7)**	8.1 (5.6 to 10.6)**	6.8 (4.5 to 9.2)**	1.7 (1.1 to 2.2)**	0.3 (0.2 to 0.5)**	0.7 (0.5 to 1.0)**
Age at adiposity peak, months (N=2489)	0.1 (-0.0 to 0.2)	0.8 (-6.1 to 7.7)	0.5 (-2.9 to 4.0)	0.6 (-2.6 to 3.8)	-0.1 (-0.8 to 0.7)	-0.1 (-0.3 to 0.1)	-0.2 (-0.5 to 0.1)

Values are linear regression coefficients (95% confidence intervals) and reflect the change in cm of childhood head circumference and in cm^3 of childhood brain structures for peak weight velocity (PWV), body mass index at adiposity peak (BMIAP) and age at adiposity peak (AGEAP). Models are adjusted for child sex, BMI and age at the neuroimaging assessment, family income and maternal age at intake, ethnicity, pre-pregnancy BMI, educational level, smoking, alcohol, and folic acid use during pregnancy. *p < 0.05. ** p < 0.01.

© 2021 Silva CCV et al. *JAMA Network Open.*

eTable 10. Associations of infant growth patterns with childhood brain outcomes (subcortical structures)

	Difference (95% Confidence Interval)						
**Characteristic**	**Thalamus volume (cm^3)**	**Amygdala volume (cm^3)**	**Hippocampus volume (cm^3)**	**Globus Pallidus volume (cm^3)**	**Putamen volume (cm^3)**	**Caudate nucleus volume (cm^3)**	**Nucleus accumbens volume (cm^3)**
Peak weight velocity, kg/y (N=2654)	0.01 (-0.01 to 0.03)	0.01 (-0.00 to 0.01)	0.01 (0.00 to 0.02)	0.00 (-0.01 to 0.01)	0.02 (-0.00 to 0.04)	-0.00 (-0.02 to 0.01)	-0.00 (-0.01 to 0.00)
BMI at adiposity peak, kg/m^2 (N=2489)	0.05 (-0.00 to 0.09)	-0.00 (-0.02 to 0.2)	0.02 (-0.01 to 0.05)	0.02 (0.00 to 0.04)*	0.08 (0.03 to 0.13)**	0.03 (-0.01 to 0.07)	-0.00 (-0.01 to 0.01)

Values are linear regression coefficients (95% confidence intervals) and reflect the change in cm of childhood head circumference and in cm^3 of childhood brain structures for peak weight velocity, BMI at adiposity peak and age at adiposity peak. Models are adjusted for child sex, intracranial volume and age at the neuroimaging assessment, family income and maternal age at intake, ethnicity, pre-pregnancy BMI, educational level, smoking, alcohol, and folic acid use during pregnancy. *p < 0.05. ** p < 0.01.

© 2021 Silva CCV et al. *JAMA Network Open.*

eTable 11. Associations of birth outcomes with childhood brain outcomes (basic models)

	Difference (95% Confidence Interval)						
**Birth outcomes**	**Head circumference (cm)**	**Total brain volume (cm^3)**	**Cerebral gray matter volume (cm^3)**	**Cerebral white matter volume (cm^3)**	**Cerebellar gray matter volume (cm^3)**	**Cerebellar white matter volume (cm^3)**	**Subcortical gray matter volume (cm^3)**
**Gestational age, week**	0.1 (0.0 to 0.1)**	6.1 (4.2 to 8.0)**	3.3 (2.4 to 4.3)**	1.7 (0.9 to 2.6)**	0.5 (0.3 to 0.7)**	0.2 (0.1 to 0.2)**	0.3 (0.2 to 0.4)**
< 37 weeks (N=138)	-0.3 (-0.5 to -0.0)*	-35.4 (-51.6 to -19.1)**	-20.3 (-28.4 to -12.2)**	-8.8 (-16.1 to -1.4)*	-3.5 (-5.3 to -1.8)**	-0.8 (-1.3 to -0.4)**	-1.8 (-2.5 to -1.1)**
37-41 weeks (N=2718)	[Reference]	[Reference]	[Reference]	[Reference]	[Reference]	[Reference]	[Reference]
>42 weeks (N=223)	0.3 (0.1 to 0.5)**	15.0 (2.0 to 28.0)*	9.1 (2.6 to 15.6)**	3.3 (-2.6 to 9.1)	1.0 (-0.3 to 2.5)	0.4 (0.0 to 0.8)*	1.1 (0.5 to 1.6)**
**Birth weight, 500g**	0.3 (0.3 to 0.4)**	24.3 (21.3 to 27.2)**	11.9 (10.4 to 13.4)**	9.1 (7.8 to 10.5)**	1.7 (1.4 to 2.0)**	0.5 (0.4 to 0.6)**	1.0 (0.8 to 1.1)**
<2500 g (N=126)	-0.7 (-1.0 to -0.5)**	-51.7 (-68.6 to -34.9)**	-24.4 (-32.8 to -16.0)**	-18.9 (-26.5 to -11.3)**	-4.2 (-6.0 to -2.4)**	-1.7 (-2.2 to -1.2)**	-2.4 (-3.2 to -1.7)**
2500-4500 g (N=2890)	[Reference]	[Reference]	[Reference]	[Reference]	[Reference]	[Reference]	[Reference]
>4500 g (N=78)	0.7 (0.4 to 1.0)**	43.6 (22.3 to 64.9)**	20.6 (9.9 to 31.2)**	19.0 (9.3 to 28.6)**	1.7 (-0.6 to 4.0)	0.6 (0.0 to 1.2)*	1.6 (0.7 to 2.6)**
**Size for gestational age, SDS**	0.4 (0.3 to 0.4)**	25.9 (22.7 to 29.1)**	12.2 (10.6 to 13.9)**	10.4 (9.0 to 11.9)**	1.8 (1.4 to 2.1)**	0.5 (0.4 to 0.6)**	0.9 (0.8 to 1.1)**
Small (<10 percentile) (N=307)	-0.7 (-0.9 to -0.5)**	-45.8 (-56.9 to -34.7)**	-21.4 (-26.9 to -15.9)**	-18.6 (-23.7 to -13.6)**	-3.1 (-4.3 to -1.9)**	-0.9 (-1.2 to -0.6)**	-1.6 (-2.1 to -1.2)**
Appropriate (10-90 percentile) (N=2458)	[Reference]	[Reference]	[Reference]	[Reference]	[Reference]	[Reference]	[Reference]
Large (>90 percentile) (N=307)	0.7 (0.5 to 0.8)**	41.2 (30.1 to 52.3)**	18.2 (12.7 to 23.8)**	17.8 (12.8 to 22.8)**	2.8 (1.6 to 4.0)**	0.8 (0.5 to 1.1)**	1.5 (1.1 to 2.0)**

Values are linear regression coefficients (95% confidence intervals) and reflect the change in cm of childhood head circumference and in cm^3 of childhood brain structures for birth outcomes. Models are adjusted for child sex and age at the neuroimaging assessment.*p < 0.05. ** p < 0.01.

© 2021 Silva CCV et al. *JAMA Network Open.*

eTable 12. Critical periods during fetal and infant life and childhood brain outcomes (basic models)

	Difference (95% CI)						
**Infant and Fetal Weight Standard Deviation Scores**	**Head circumference (cm)**	**Total brain volume (cm^3)**	**Cerebral gray matter volume (cm^3)**	**Cerebral white matter volume (cm^3)**	**Cerebellar gray matter volume (cm^3)**	**Cerebellar white matter volume (cm^3)**	**Subcortical gray matter volume (cm^3)**
At 20 weeks	0.1 (0.0 to 0.2)**	6.0 (1.4 to 10.5)**	3.1 (0.8 to 5.4)**	2.3 (0.2 to 4.4)*	0.3 (-0.2 to 0.8)	0.1 (-0.1 to 0.2)	0.2 (-0.0 to 0.4)
At 30 weeks	0.3 (0.2 to 0.3)**	16.1 (11.8 to 20.5)**	7.7 (5.5 to 9.9)**	6.5 (4.5 to 8.5)**	1.0 (0.6 to 1.5)**	0.4 (0.3 to 0.5)**	0.5 (0.3 to 0.7)**
At birth	0.4 (0.3 to 0.4)**	23.2 (18.8 to 27.5)**	10.8 (8.6 to 13.0)**	9.4 (7.4 to 11.4)**	1.6 (1.1 to 2.1)**	0.4 (0.3 to 0.6)**	0.9 (0.7 to 1.1)**
At 6 months	0.4 (0.3 to 0.4)**	12.9 (8.5 to 17.2)**	5.2 (3.0 to 7.4)**	5.4 (3.4 to 7.4)**	1.1 (0.7 to 1.6)**	0.4 (0.3 to 0.5)**	0.7 (0.5 to 0.8)**
At 12 months	0.3 (0.3 to 0.4)**	12.3 (7.9 to 16.6)**	4.8 (2.6 to 7.0)**	5.3 (3.3 to 7.3)**	1.3 (0.9 to 1.8)**	0.4 (0.3 to 0.6)**	0.4 (0.2 to 0.6)**
At 24 months	0.3 (0.2 to 0.4)**	11.7 (7.3 to 16.0)**	6.3 (4.1 to 8.5)**	4.0 (2.0 to 6.0)**	0.7 (0.2 to 1.2)**	0.3 (0.2 to 0.4)**	0.4 (0.2 to 0.6)**

Values are linear regression coefficients (95% confidence intervals) from conditional analyses and reflect the change in cm of childhood head circumference and in cm^3 of childhood brain structures for fetal and infant weight. Models are adjusted for child sex and age at the neuroimaging assessment. *p < 0.05. ** p < 0.01.

© 2021 Silva CCV et al. *JAMA Network Open.*

eTable 13. Associations of longitudinal fetal and infant growth patterns with childhood brain outcomes (basic models)

	Difference (95% Confidence Interval)						
**Growth patterns**	**Head circumference (cm)**	**Total brain volume (cm^3)**	**Cerebral gray matter volume (cm^3)**	**Cerebral white matter volume (cm^3)**	**Cerebellar gray matter volume (cm^3)**	**Cerebellar white matter volume (cm^3)**	**Subcortical gray matter volume (cm^3)**
**Fetal growth deceleration**							
Infant growth deceleration (N= 80)	-0.8 (-1.2 to -0.5)**	-31.6 (-53.0 to -10.2)**	-15.9 (-26.6 to -5.2)**	-10.3 (-20.0 to -0.5)*	-3.6 (-5.9 to -1.3)**	-0.8 (-1.5 to -0.2)*	-0.8 (-1.8 to 0.1)
Infant normal growth (N= 245)	-0.3 (-0.5 to -0.1)*	-14.5 (-28.3 to -0.7)*	-7.2 (-14.1 to -0.3)*	-4.4 (-10.7 to 1.9)	-1.8 (-3.3 to -0.3)*	-0.4 (-0.8 to 0.0)	-0.6 (-1.2 to -0.0)*
Infant growth acceleration (N= 265)	0.1 (-0.1 to 0.3)	-11.3 (-24.7 to 2.1)	-5.7 (-12.4 to 1.1)	-5.0 (-11.2 to 1.1)	-0.3 (-1.8 to 1.2)	0.1 (-0.3 to 0.5)	-0.5 (-1.1 to 0.1)
**Fetal normal growth**							
Infant growth deceleration (N= 210)	-0.2 (-0.4 to 0.1)	-4.7 (-19.2 to 9.9)	0.0 (-7.3 to 7.3)	-2.9 (-9.5 to 3.8)	-1.3 (-2.8 to 0.3)	-0.3 (-0.8 to 0.1)	-0.2 (-0.9 to 0.4)
Infant normal growth (N= 532)	[Reference]	[Reference]	[Reference]	[Reference]	[Reference]	[Reference]	[Reference]
Infant growth acceleration (N= 274)	0.5 (0.3 to 0.7)**	7.3 (-6.0 to 20.6)	2.5 (-4.2 to 9.1)	4.2 (-1.9 to 10.3)	-0.2 (-1.7 to 1.2)	0.4 (0.0 to 0.8)*	0.4 (-0.2 to 1.0)
**Fetal growth acceleration**							
Infant growth deceleration (N= 304)	0.2 (-0.0 to 0.4)	22.7 (9.9 to 35.6)**	10.7 (4.3 to 17.1)**	9.3 (3.5 to 15.2)**	1.5 (0.1 to 2.9)*	0.5 (0.1 to 0.9)**	0.7 (0.1 to 1.2)*
Infant normal growth (N= 313)	0.6 (0.4 to 0.8)**	35.1 (22.3 to 47.8)**	17.5 (11.1 to 23.9)**	13.9 (8.1 to 19.7)**	1.6 (0.2 to 2.9)*	0.7 (0.3 to 1.1)**	1.3 (0.7 to 1.8)**
Infant growth acceleration (N= 94)	1.3 (0.9 to 1.6)**	35.9 (15.9 to 55.9)**	15.3 (5.3 to 25.3)**	18.2 (9.1 to 27.4)**	0.1 (-2.0 to 2.3)	0.9 (0.3 to 1.5)**	1.4 (0.5 to 2.2)**

Values are linear regression coefficients (95% confidence intervals) and reflect the change in cm of childhood head circumference and in cm^3 for each childhood brain structures compared to children with normal fetal and infant growth. Models are adjusted for child sex and age at the neuroimaging assessment.*p < 0.05. ** p < 0.01.

© 2021 Silva CCV et al. *JAMA Network Open.*

eTable 14. Associations of infant growth patterns with childhood brain outcomes (basic models)

	Difference (95% Confidence Interval)						
**Characteristic**	**Head circumference (cm)**	**Total brain volume (cm^3)**	**Cerebral gray matter volume (cm^3)**	**Cerebral white matter volume (cm^3)**	**Cerebellar gray matter volume (cm^3)**	**Cerebellar white matter volume (cm^3)**	**Subcortical gray matter volume (cm^3)**
Peak weight velocity, kg/y (N=2654)	0.2 (0.2 to 0.2)**	4.5 (2.6 to 6.4)**	1.5 (0.5 to 2.4)**	2.2 (1.4 to 3.1)**	0.4 (0.2 to 0.6)**	0.2 (0.1 to 0.3)**	0.2 (0.1 to 0.3)**
BMI at adiposity peak, kg/m^2 (N=2489)	0.5 (0.5 to 0.6)**	16.4 (11.6 to 21.2)**	7.1 (4.7 to 9.5)**	6.8 (4.6 to 9.0)**	1.1 (0.6 to 1.6)**	0.6 (0.5 to 0.7)**	0.7 (0.5 to 0.9)**
Age at adiposity peak, months (N=2489)	0.1 (0.0 to 0.2)*	6.9 (-0.2 to 14.0)	3.7 (0.2 to 7.2)*	2.6 (-0.6 to 5.9)	0.5 (-0.2 to 1.3)	0.0 (-0.2 to 0.2)	0.0 (-0.3 to 0.3)

Values are linear regression coefficients (95% confidence intervals) and reflect the change in cm^3 of childhood brain structures for peak weight velocity, BMI and age at adiposity peak. Models are adjusted for child sex and age at the neuroimaging assessment. *$p < 0.05$. ** $p < 0.01$.

© 2021 Silva CCV et al. *JAMA Network Open.*
